# Liver X Receptors: A Possible Link between Lipid Disorders and Female Infertility

**DOI:** 10.3390/ijms19082177

**Published:** 2018-07-25

**Authors:** Sarah Dallel, Igor Tauveron, Florence Brugnon, Silvère Baron, Jean Marc A. Lobaccaro, Salwan Maqdasy

**Affiliations:** 1 Université Clermont Auvergne, GReD, CNRS UMR 6293, INSERM U1103, 28, Place Henri Dunant, BP38, F63001 Clermont-Ferrand, France; sarah.dallel@etu.uca.fr (S.D.); itauveron@chu-clermontferrand.fr (I.T.); smaqdasy@chu-clermontferrand.fr (S.M.); 2 Centre de Recherche en Nutrition Humaine d’Auvergne, 58 Boulevard Montalembert, F-63009 Clermont-Ferrand, France; 3 Service d’Endocrinologie, Diabétologie et Maladies Métaboliques, CHU Clermont Ferrand, Hôpital Gabriel Montpied, F-63003 Clermont-Ferrand, France; 4 Université Clermont Auvergne, ImoST, INSERM U1240, 58, rue Montalembert, BP184, F63005 Clermont-Ferrand, France; fbrugnon@chu-clermontferrand.fr; 5 CHU Clermont Ferrand, Assistance Médicale à la Procréation-CECOS, Hôpital Estaing, Place Lucie et Raymond Aubrac, F-63003 Clermont-Ferrand CEDEX 1, France

**Keywords:** liver X receptors, cholesterol, female reproduction, breast cancer, ovarian hyperstimulation syndrome

## Abstract

A close relationship exists between cholesterol and female reproductive physiology. Indeed, cholesterol is crucial for steroid synthesis by ovary and placenta, and primordial for cell structure during folliculogenesis. Furthermore, oxysterols, cholesterol-derived ligands, play a potential role in oocyte maturation. Anomalies of cholesterol metabolism are frequently linked to infertility. However, little is known about the molecular mechanisms. In parallel, increasing evidence describing the biological roles of liver X receptors (LXRs) in the regulation of steroid synthesis and inflammation, two processes necessary for follicle maturation and ovulation. Both of the isoforms of LXRs and their bona fide ligands are present in the ovary. *LXR*-deficient mice develop late sterility due to abnormal oocyte maturation and increased oocyte atresia. These mice also have an ovarian hyper stimulation syndrome in response to gonadotropin stimulation. Hence, further studies are necessary to explore their specific roles in oocyte, granulosa, and theca cells. LXRs also modulate estrogen signaling and this could explain the putative protective role of the LXRs in breast cancer growth. Altogether, clinical studies would be important for determining the physiological relevance of LXRs in reproductive disorders in women.

## 1. Introduction

Infertility affects 15% of couples and remains one of the major issues of public health. The propagation of infertility from the last forty years [1] seems parallel to the increment of the prevalence of obesity, dyslipidemia, and metabolic syndrome [2,3]. A strong correlation exists between the lipid metabolism and the reproductive function in both men and women [4,5,6,7,8]. Cholesterol influences female fertility through multiple mechanisms, especially oocyte maturation, cell turnover during folliculogenesis, and hormonal function of the ovary and placenta. Indeed, a tight control of cholesterol turnover in the steroidogenic tissues is mandatory in order to maintain a normal ovarian function. Hence, an excess or insufficiency of cholesterol is deleterious for cell function. The turnover starts from the cholesterol uptake through low density lipoprotein (LDL) and high density lipoprotein (HDL) receptors (LDLR and SR-BI, respectively), cholesterol esterification and storage, cholesterol ester hydrolase activity, cholesterol de novo synthesis pathway, and cholesterol efflux promoted by ATP-binding cassette (ABC) proteins. The latter are target genes of liver X receptors.

Liver X receptors (LXRα/NR1H3 and LXRβ/NR1H2) are nuclear receptors (NRs) for oxysterols, which are largely implicated in cholesterol homeostasis [9]. Because of their roles in the regulation of numerous metabolic functions, LXRs could represent part of the molecular link between lipid disorders and infertility. Thus, many arguments led to deeply investigating LXRs in female reproductive function, namely: (1) the close relationship between cholesterol and ovarian physiology [4,10,11]; (2) the increasing body of literature describing the novel biological roles of LXRs in the regulation of steroid synthesis and inflammation, two processes necessary for follicle maturation and ovulation [12,13,14,15,16,17]; (3) the fact that oxysterols, which are the physiological ligands [18], play a potential role in the maturation of the oocytes [19,20]; (4) and the role of LXRs in spermatogenesis suggests common germ cell pathways regulated by LXRs [7,14,17].

## 2. Fertility Disorders and Abnormal Lipid Homeostasis

Ovarian dysfunction is frequently observed in patients with metabolic syndrome and obesity, two frequent pathological situations linked to polycystic ovary syndrome (PCOS) [21,22], which is the main cause of amenorrhea and infertility in women [23,24,25]. These women share the main metabolic anomalies related to overweight and insulin resistance [26]. Indeed, 50–70% of women who suffer from PCOS are obese and 43% of them suffer from metabolic syndrome characterized by low HDL levels [6]. These patients also present inefficient folliculogenesis [27]. Furthermore, glycation end products in obese patients affect granulosa cell physiology counteracting a LH effect on such cells, and inducing the inflammatory cytokine secretion responsible for anovulation [28]. Furthermore, the oocytes of women with PCOS are smaller.

The Danish register of 47,000 couples identified a link between body mass index and infertility [29]. Moreover, the chances of success of assisted reproduction techniques to obtain a pregnancy are reduced in these women. They are resistant to gonadotrophin stimulation. The ovarian dysfunction in obese women is reflected by lower anti-Mullerian hormone (AMH) and inhibin B levels, two markers of granulosa cells reflecting the ovarian follicular reserve and the endocrine activity of the ovary [30,31]. The association between obesity and infertility seems to be correlated, at least in part, to anomalies in lipid metabolism [21]. Indeed, free cholesterol levels are positively correlated with the mean duration necessary to become pregnant [4].

Actually, the molecular evidences linking cholesterol anomalies and infertility came from mouse models. Cholesterol-rich diet alters the oocyte quality and reduces the ovulation rate in mice [32]. The follicles of these mice are apoptotic [33]. Furthermore, the oxidative stress seems to be higher in the oocytes of obese mice [34]. At the molecular level, the female mice deficient for *Abca1* encoding the ATP-binding cassette, A1, involved in cholesterol efflux, have low HDL levels and suffer from steroidogenesis defects, a reduced number of pups per litter, and placenta anomalies [10]. Likewise, the mice deficient for *Srb1* encoding the scavenger receptor class B type 1 (SRB1/SCARB1), also known as HDL-receptor, are infertile, with lower cholesteryl ester levels in the ovary and with defects in embryogenesis and implantation [35]. Conversely, excessive free cholesterol could also affect the meiosis in *Srb1−/−* or in wild type mice fed a cholesterol-rich diet [11]. The oocyte of *Srb1−/−* mice skips meiosis arrest and expulses spontaneously its second germinal vesicle, explaining the sterility in such mice.

Apolipoprotein E (Apoe)-deficient mice have a lower expression of *Cyp19a1*, encoding aromatase, and *Hsd3b*, encoding 3 β-hydroxysteroid dehydrogenase/∆5-4-isomerase, which catalyzes the conversion of pregnenolone to progesterone, and the oxidative conversion of other ∆5-ene-3-beta-hydroxy steroid. The folliculogenesis is enhanced, however, it is counteracted by excessive follicular atresia. Paradoxically, no modification in their fertility rates has been identified [36].

The analysis of infertile women revealed polymorphisms on SCARB1, consolidating the link between cholesterol, its receptor, and fertility. In another study, the HDL and apolipoprotein (APO) A-1 levels in the follicular fluid obtained during in vitro fertilization were negatively correlated with the embryonic development in the early stages [37]. Likewise, the APOE polymorphisms alter the plasma cholesterol and apolipoprotein levels [38], and are associated with reduced reproductive efficiency in women with *APOE2* protein subtype [39].

In summary, cholesterol metabolism anomalies are directly linked to oocyte maturation and to chances of fertility. Because various nuclear receptors (NRs) are involved in the control of cholesterol homeostasis, many research groups have looked for a putative implication of these transcription factors in the control of the female fertility. If the ‘classical’ steroid NRs, such as those of progesterone (PR/NR3C3), estrogens (ERα/NR3A1 and ERβ/NR3A2), and androgens (AR/NR3C4), have been extensively studied, the ‘lipid’ NRs, such as LXRs, FXR (bile acid receptor; NR1H4), SHP (small heterodimeric partner; NR0B1), and LRH1 (liver receptor homolog 1; NR5A2), have been the topic of investigations, mainly because of the phenotypes observed in the mice lacking the genes encoding these NRs. We will focus this review on LXRs.

## 3. LXRs and Their Ligands in the Ovary

LXRα and LXRβ are two NRs whose natural ligands and activators are derived from specific oxidized forms of cholesterol [18,40], or dendrogenin A, the product of a stereo-selective condensation of 5,6α-epoxycholesterol with histamine [41]. The discovery of this ligand identified the existence of a new metabolic branch at the crossroad between cholesterol and histamine metabolism [42].

**Figure 1:**
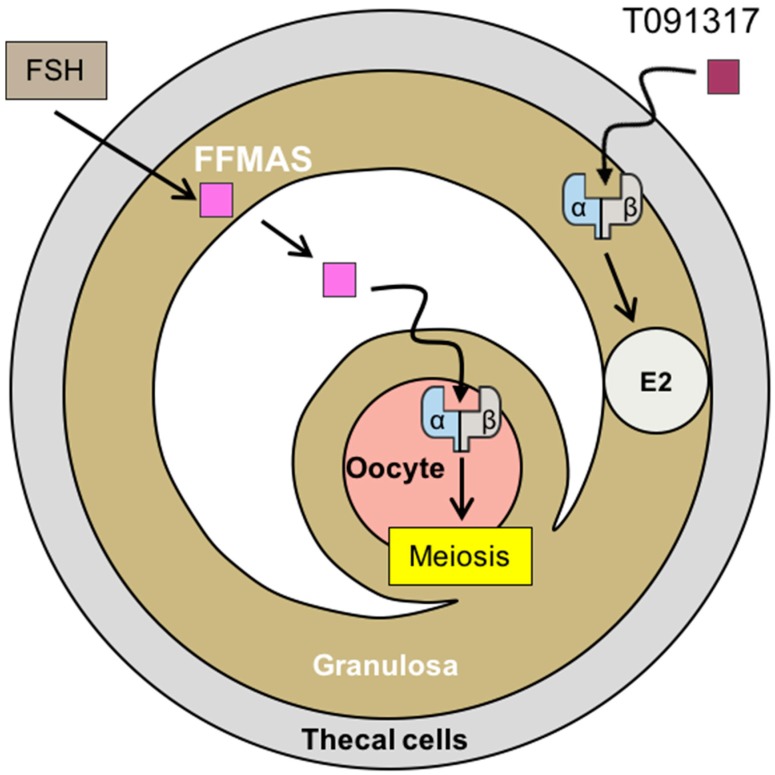
Role of liver X receptors (LXRs) in oocyte meiosis and in estradiol synthesis. When follicle-stimulating hormone (FSH )reaches its receptor on the granulosa cells, it increases the concentration of follicular fluid meiosis-activating sterol (FFMAS) by increasing its synthesis, a ligand of LXRα/β. This in turn induces the final steps of the oocyte meiosis. In addition, when the LXRα/β is activated by a ligand (in this figure T0901317, a synthetic ligand, purple square), they increase the production of estradiol. α/β—LXRα, or LXRβ; E2—estradiol; FFMAS—follicular fluid meiosis-activating sterol (pink square).

Both of the isoforms are found in the oocyte with a predominance of LXRβ [16,20]. This expression is induced by the human chorionic gonadotropin hormone (hCG), and plays an important role in steroidogenesis in humans [43] as well as mice [16]. Follicular fluid meiosis-activating sterol (FFMAS), which can activate LXRs, increases after stimulation by gonadotropins [44,45] (Figure 1). This increment is necessary for the oocyte to resume meiosis just before ovulation. Indeed, the luteinizing hormone (LH) surge during folliculogenesis, necessary for ovulation, induces meiosis resumption of the oocyte. This indirect effect is mediated by the FFMAS produced by granulosa cells. Thus, FFMAS stimulates its receptor on the oocytes and LXRα was suggested as a candidate [18,40]. Furthermore, FFMAS promotes embryo implantation [46].

**Figure 2:**
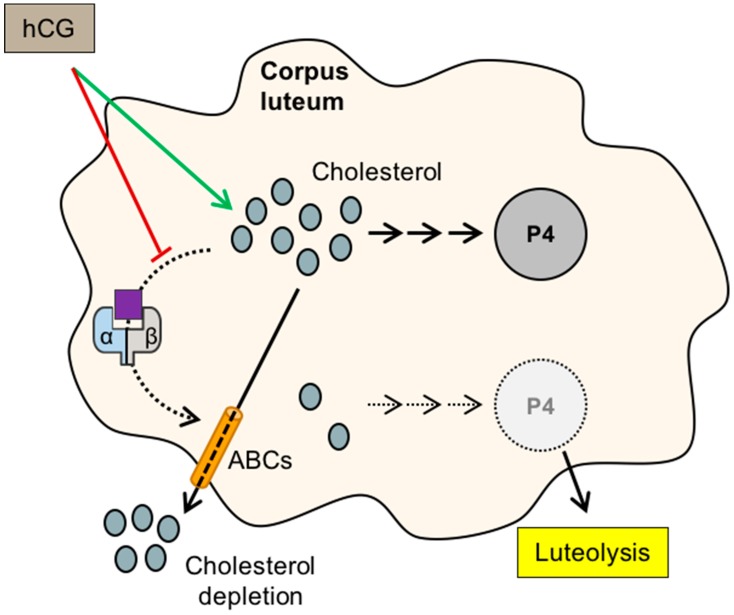
Role of LXRs in progesterone production and luteolysis. When the human chorionic gonadotropin hormone (hCG) reaches its receptor, it increases (green arrow) the concentration of cholesterol, by acting on low density lipoprotein receptor (LDLR) (uptake) and sterol response binding element (SREBP2) (de novo synthesis), and favors the production of progesterone (P4). Activation of LXRα/β by one of their bona fide ligands, produced from the cholesterol oxidation, stimulates the production of ATP-binding cassette transporter (ABC) proteins, inducing a cholesterol depletion within the cell, a decrease in progesterone synthesis, and finally, the luteolysis. hCG also inhibits LXR transcriptional activity. α/β—LXRα or LXRβ; ABCs—ATP-binding cassette transporters; hCG—human chorionic gonadotropin; P4—progesterone. LXR ligands are represented by the purple square.

During the luteal phase, LXR is thought to promote luteolysis by depriving the lutein cells from cholesterol, a key molecule for progesterone synthesis (Figure 2). This effect is counteracted by the activity hCG that inhibits the LXR activity in these cells, and increases the sterol response element binding protein 2 and LDLR expression to maintain a cholesterol supply [47].

Steffensen et al. [20] first described the phenotype of female mice lacking both LXRs. These mice are hypo fertile with a reduced number of pups per litter. This seems mainly due to the absence of LXRβ. With their folliculogenesis and ovulation being normal, these mice suggest putative defects in oocyte maturation and/or in meiosis resumption [46]. LXRs are thus intermediates that promote the action of gonadotropins on the oocyte. In the same line of evidence, Grondahl et al. tested ex vivo meiosis resumption of oocytes surrounded by their cumulus using FFMAS and the other LXR ligands, 22R-hydroxycholesterol, 16-hydroxycholesterol, 25-hydroxycholesterol, and 27-hydroxycholesterol. Meiosis resumption was induced only with FFMAS [19]. Conversely, Steffensen et al. showed that zymosterol, an intermediate in cholesterol biosynthesis and the structural analog of FFMAS, is capable of inducing meiosis resumption, however, in a LXR-dependent manner. In wild type mice, an FSH injection induces meiosis resumption and increases the *Lxrα* transcription levels. On the other side, the synthetic LXR agonist GW3965 induces meiosis resumption, even in the absence of the cumulus, suggesting that LXR in the oocyte is necessary for its maturation [20]. On the cumulus–oocyte complex retrieved from *Lxr−/−* mice, the meiosis resumption was abolished despite a treatment with FSH, zymosterol, or GW3965 [20]. These findings suggest that FSH activates the FFMAS production from the cumulus, which then activates the LXR in the oocyte. Likewise, our team pointed out that deficient-LXR mice have a delayed sterility and an inefficient folliculogenesis under stimulation, with an important number of atretic oocytes on retrieval, after stimulation by gonadotropins [16]. Altogether, LXRs are important for oocyte maturation and survival.

As in the testes [14,17] and adrenal glands [12], LXRs are also involved in the regulation of steroid synthesis in the ovary [16]. Indeed, the synthetic LXR ligand T0901317 induces the estradiol synthesis in wild type mice by activating the transcription of the steroidogenic acute regulatory protein (StAR), a transport protein that regulates cholesterol transfer within the mitochondria, which is the rate-limiting step in the production of steroid hormones. In the ovary, LXRs also control the transcription of cytochrome P450 side-chain cleavage (Cyp11A1), a mitochondrial enzyme that catalyzes the conversion of cholesterol to pregnenolone, the first reaction in the process of steroidogenesis.

Interestingly, LXRs seem to control any excessive estradiol synthesis during gonadotropin stimulation. Indeed, the gonadotropin stimulation of the *Lxr−/−* mice leads to an exaggerated hormonal response, with an excessive estradiol secretion [16]. This effect contributes to the phenotype of ovarian hyper stimulation syndrome (OHSS), observed in LXR-deficient mice. After a gonadotropin stimulation, the ovaries of the *Lxr−/−* mice harvest large hemorrhagic follicles with an exaggerated inflammatory and ovulatory response. This signals a diagnosis of OHSS, which is characterized by the excessive accumulation of vasoactive and angiogenic substances activating the vascular epithelial growth factor (VEGF) and interleukin (IL) 6 signaling, leading to a systemic inflammatory response, and variable clinical manifestations of cardiovascular collapse, septic shock, and thromboembolism in the most severe cases [48,49,50,51,52,53].

Although VEGF levels and increased IL6 signaling (through its receptor sIL-6Rα) are linked to OHSS, no molecular driver has been identified yet. Furthermore, the literature information are contradictory in linking these two cytokines to OHSS [54]. As LXRs have anti-inflammatory properties from inhibiting IL6, COX2, tumor necrosis factor TNF*α* [55], and the downstream of VEGF signaling [56], they could potentially be implicated in the prevention of OHSS [13,57,58,59]. Altogether, the genetic models enlighten the LXRs as key factors for the endocrine and exocrine functions of the ovary, and suggest that these NRs could be considered gatekeepers against an exaggerated ovarian response to gonadotropins. Even though clinical investigations should be performed, the exact roles of LXRs in femal ovarian physiology is thus questioned.

## 4. LXRs, Uterus, and Placenta

The endometrium trophicity is influenced by estrogens and progesterone in each menstrual cycle, in to be prepared for eventual pregnancy. When implantation takes place, the placenta develops in the endometrium permitting maternofetal exchange during pregnancy. Myometrium is necessary during labor. Obesity is related to many complications associated with pregnancy (e.g., gestational diabetes mellitus, tromboembolic problems, and hypertensive disorders such as preeclampsia or eclampsia) [60]. Hence, obese patients and/or those with a metabolic syndrome usually suffer from dystocia, contractility defects during labor that could affect the perinatal morbidity, and mortality [61,62]. Likewise, before pregnancy, the body mass index [63,64] and its increase [65] during pregnancy have been associated with a higher risk for caesarean delivery at the term of pregnancy, for failure to progress in the labor.

**Figure 3:**
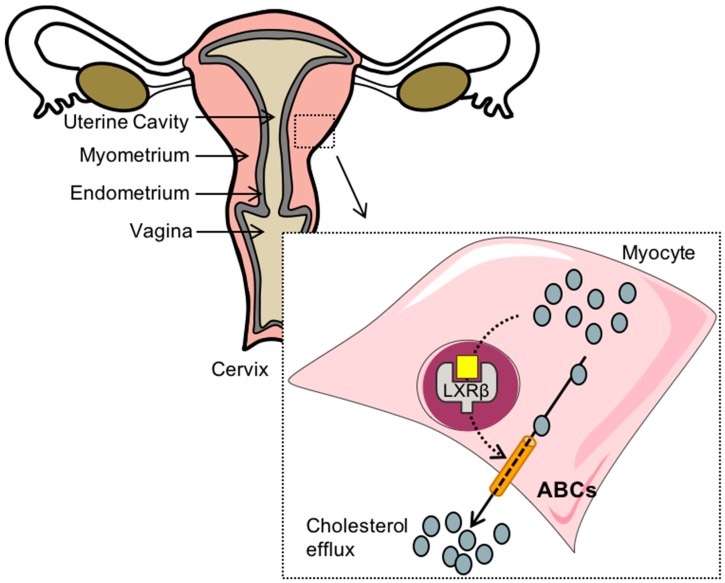
LXRβ controls the cholesterol homeostasis within the myometrium. When cholesterol (grey) raises to a critical concentration, its enzymatic transformation into LXR-activating oxysterols induces a higher accumulation of ATP-binding caste transporters and the efflux of cholesterol. A defect in the LXRβ-signaling pathway is linked to a higher accumulation of cholesteryl esters, a decrease response to oxytocin and prostaglandin (PG) F2α, and a defect in the contractility during the labor. ABCs—ATP-binding cassette transporters; LXRβ—liver X receptor β. Oxysterols are represented by the yellow square.

Interestingly rats fed a hypercaloric diet to induce obesity have an increased accumulation of transcripts implicated in lipid homeostasis and inflammation, such as fatty acid translocase CD36, lipoprotein lipase, and nuclear factor kappa-light-chain-enhancer of activated B cells (NF-κB), in the uterus [66]. This accumulation is also observed in ob/ob and db/db mouse models, which are prone to develop obesity as well as metabolic syndrome [67]. An abnormal accumulation of cholesterol in the muscular cells of the myometrium has been associated to contractility defects [68,69]. We showed that the females lacking LXRβ presented a similar phenotype of characterized by a higher accumulation in the myometrium, resulting from the absence of the upregulation of *Abca1* and *Abcg1* (Figure 3). Together with this ‘adipose’-like phenotype of the muscular cells, the LXRβ-deficient mice exhibited a lower capacity to contract under stimulation with oxytocin and prostaglandin (PG) F2α analog [70]. This link between lipid accumulation in the myometrium and resistance to oxytocin induction during labor was suggested by Andreasen et al., who described a need for more oxytocin infusion to induce labor in overweight and obese women than in normal weight [60].

Nutrient and hormone exchanges between maternal and fetal circulation take place through the placenta, which also constitutes a barrier against toxins, in order to protect the fetus. Cholesterol is supplied by maternal circulation for steroid synthesis, by the placenta [71]. Among the various metabolic disorders during pregnancy, preeclampsia is one of the most important and affects 5% of pregnant women [72,73]. This pathology usually occurs in women with obesity and diabetes, and is characterized by hypertension and proteinuria [74,75]. The hallmark of this syndrome is an insufficient trophoblast invasion with contractile spiral uterine arteries, leading to acute atherosis (atherosclerosis-like), vasoconstriction, and hypertension [75,76]. Elevated oxidized LDL (rich in sterols) levels reduce vessel invasion and produce preeclampsia [77]. Early and progressive LXRα and LXRβ expression in the placenta (seven days post-coitum in mice, six weeks of pregnancy in women) participate to maintain the cholesterol available for trophoblast cells, and modulates arterial invasion [78,79]. Many oxysterols, especially 25-hydroxycholesterol, increase in the placenta during pregnancy [80]. Furthermore, LXRs activate the ABC protein expression in trophoblast cells for eliminating excessive cholesterol and toxic sterols [78,81]. LXRβ also controls trophoblast invasion [77,82,83]. Interestingly, the *LXRα* and *ABCA1* genes are overexpressed in the placenta tissue of the women that suffered from preeclampsia [79], while another study demonstrated a reduction in LXR*β* in these patients [84]. Endoglin/CD105 is membrane receptor that induces endothelial relaxation [78,85,86]. The production of soluble Endoglin/CD105 by the membrane metalloproteinase-14 induces the squelching of TGF-β1, endothelial dysfunction, and impaired relaxation, altogether, preeclampsia [78,85,86]. We pointed out that Endoglin/CD105 is an atypical LXR target gene, which could explain how LXR could reduce the trophoblast invasion and the risk of preeclampsia [78,85,86]. Interestingly, a single nucleotide polymorphism within the sequences encoding LXRβ has been significantly associated with the risk of preeclampsia in a study of over 155 women that presented this disorder [87].

## 5. LXRs, Modulation of Estrogen Activity, and Breast Cancer

Even though breast cancer cannot be defined as a woman reproductive disease per se, it has been directly associated to the circulating levels of estrogens and the levels and/or mutations of ERα by their roles in the growth and proliferation of epithelial cells. Hence, pharmacological management partly targets the estrogen pathway by using selective estrogen receptor modulators (SERM; e.g., tamoxifen or raloxifen) or degraders (SERD; e.g., fulvestran), LHRH analogs, and/or aromatase inhibitors [88,89]. Nevertheless, these therapies induce menopausal symptoms and some breast cancers are negative for ERα [90]. A significant correlation between ERα positive breast cancer/obesity/metabolic syndrome from one side, and statin, an inhibitor of 3-hydroxy-3-methylglutaryl CoA reductase and cholesterol de novo synthesis, from the other side, have been revealed by various studies [91,92,93,94]. Beside the excessive aromatization of estrogen by the adiposity, and the excessive production of insulin-like growth factors and inflammatory cytokines [95], LXRs could represent a molecular link. Indeed, LXRs and ERα exert reciprocal effects on each other. As mentioned above, LXRs are gatekeepers against excessive E2 production after hormonal stimulation. LXR activation reduces ERα expression [96]. Furthermore, LXRs decrease the free estrogen level by increased sulfotransferase activity (EST or SULT1E1) [97]. On the other side, E2 decreases the LXR mRNA expression [98,99,100,101]. The ERα in the liver is recruited to the SREBP1c promoter, through direct binding to LXR, and prevents coactivator recruitment to LXR in an estrogen dependent manner [100].

**Figure 4:**
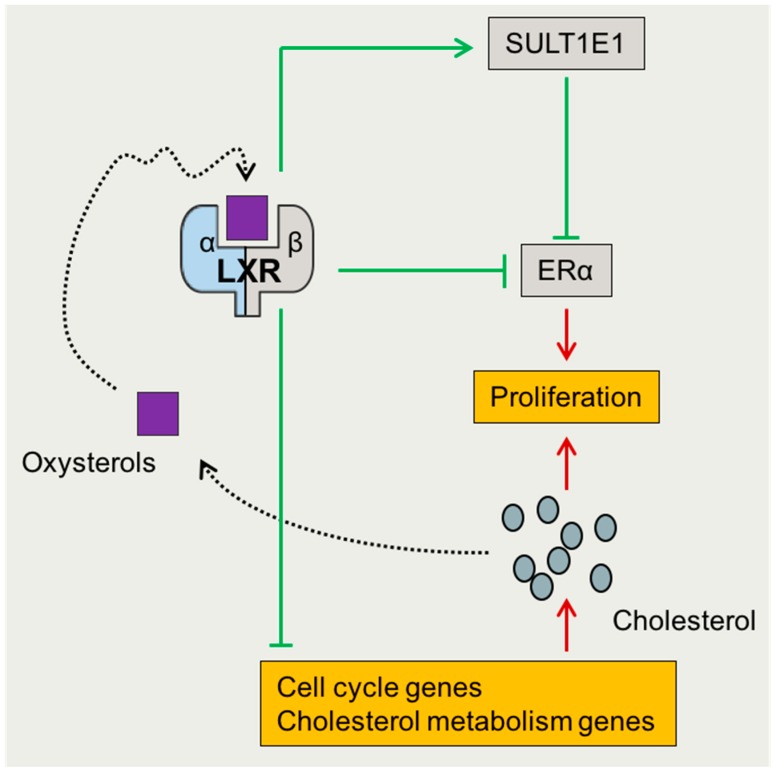
LXRs have beneficial effects on breast cancer proliferation. While increased synthesis and/or concentration of cholesterol induce cell proliferation, activation of LXRs blocks estrogen receptor (ER) α transcriptional effects directly by decreasing its transcription and by increasing sulfotransferase Family 2A Member 1 (SULT1A1), which in turn decreases the levels of circulating estrogens. ERα—estrogen receptor α; LXRα/β—liver X receptor α/β; SULT1E1—sulfotransferase E1.

LXRs were also identified to have an anti-proliferative effect in both ER positive and ER negative cells lines [96,102]. They could block cell proliferation-invasion by downregulating the cell cycle and cholesterol metabolism genes. The hypothesis of the LXR protective effect, through its anti-proliferative, pro-apoptotic role is based on cholesterol deprivation, as cholesterol is indispensable for cell proliferation [103,104,105]. Furthermore, the LXR activation reduced different breast cancer cell lines in vitro, by the suppression of cyclin proteins, ERα, and increased P53 protein levels [96]. LXR manipulation could help in estrogen deprivation, necessary for breast cancer treatment [106]. The molecular mechanism seems to be linked to LXRβ, which inhibits the proliferation of human breast cancer cells through the PI3K–Akt pathway [107] or an E2F-mediated mechanism [102,107]. Altogether, this thus indicates a positive impact of LXRs for protection against the development of breast cancer (Figure 4), even though 27-hydroxycholesterol, which is a LXR-ligand, acts as ERα ligand SERM [108] in breast cancer, cannot exclude a negative role of LXRs when 27-hydroxycholesterol increases, which has never been described so far. Despite this possibility, modulating the cholesterol levels by statin treatment, or LXRs agonists, has been evoked as potential therapeutic option to prevent and/or repress tumor growth [109].

## 6. Conclusions

This review highlights the roles of both LXRs in female physiology (oocyte maturation, fertility, and delivery) and the potential abnormal regulations of their signaling pathways in female pathophysiology. Reduced LXR activity could indeed expose the oocyte to higher cholesterol and estradiol concentrations, and alter the oocyte meiosis and survival. Furthermore, LXRs are gatekeepers against ovarian hyper stimulation. LXR signaling could be a perspective for future clinical studies to identify women at high risk of OHSS development, as well as how LXR modulation in parallel to the protocols of hormone stimulation during procreation assistance could abolish an excessive response. The beneficial role of LXRs in breast cancer has been suggested from genetic models and cell lines. More clinical investigations will be necessary before translating these findings to the clinics. Moreover, the fact that LXRs are ubiquitous transcription factors with pleiotropic physiological activities makes it mandatory to identify selective LXR modulators (SLiMs), as this was done for the SERMs and ER. For that, new pharmacology paradigms will be necessary as well.

## Abbreviations

ABCA1/G1	ATP-binding cassetteA1/G1
AMH	anti-Mullerian hormone
APO	apolipoprotein
COX	cyclooxygenase
Cyp11A1	cytochrome P450 side-chain cleavage
Cyp19a1	cytochrome P450 aromatase
ER	estrogen receptor
EST	estrogen sulfotransferase
FFMAS	follicular fluid meiosis-activating sterol
hCG	human chorionic gonadotropin
HDL	high density lipoprotein
Hsd3b	3 β-hydroxysteroid dehydrogenase/∆5-4-isomerase
IL6	interleukin 6
iNOS	inducible nitric oxide synthase
JAK/STAT	janus kinase/signal transducer and activator of transcription protein
LDL	low density lipoprotein
LDLR	LDL receptor; LXR, liver X receptor
*Lxr−/−* mice	LXR-deficient mice
NR	nuclear receptor
OHSS	ovarian hyper stimulation syndrome
PCOS	polycystic ovary syndrome
PI3K	phosphoinositide 3-kinase
SCARB1	scavenger receptor class B type
SREBP	sterol response element binding protein
SLiMs	selective liver X receptor modulators
StAR	steroidogenic acute regulatory protein
SULT2A1	Sulfotransferase Family 2A Member 1
VEGF	vascular endothelial growth factor
27OHC	27-hydroxycholesterol
